# Gov➔Value: How to combine reported quality experiences and patient-reported outcome measures. First results on vulvar cancer patients in an Italian Research Hospital

**DOI:** 10.3389/fpubh.2022.1014651

**Published:** 2022-12-20

**Authors:** Egidio de Mattia, Carmen Angioletti, Alessio Perilli, Linda Stella Guajardo Rios, Giorgia Garganese, Luca Tagliaferri, Giovanni Scambia, Simona Maria Fragomeni, Antonio Giulio de Belvis

**Affiliations:** ^1^Clinical Pathways and Outcomes Evaluation Unit, Fondazione Policlinico Universitario ‘A. Gemelli’—IRCCS, Rome, Italy; ^2^Section of Hygiene, Department of Life Sciences and Public Health, Università Cattolica del Sacro Cuore, Rome, Italy; ^3^Department of Woman and Child Health and Public Health, Fondazione Policlinico Universitario ‘A. Gemelli’—IRCCS, Rome, Italy; ^4^Gynecology and Breast Care Center, Mater Olbia Hospital, Olbia, Italy; ^5^Section of Gynecology and Obstetrics, Department of Life Sciences and Public Health, Università Cattolica del Sacro Cuore, Rome, Italy; ^6^Unit of Oncological Radiotherapy, Department of Diagnostic Imaging, Oncological Radiotherapy and Hematology, Fondazione Policlinico Universitario ‘A. Gemelli’—IRCCS, Rome, Italy; ^7^Unit of Cancer Gynecology, Department of Woman and Child Health and Public Health, Fondazione Policlinico Universitario ‘A. Gemelli’—IRCCS, Rome, Italy

**Keywords:** patient-reported outcome measures (PROM), healthcare quality, oncologic care, value based healthcare, audit & feedback

## Abstract

**Introduction:**

Vulvar cancer (VC) accounts for <1% of cancers affecting the female gender. Clinical Pathways (CP) and Clinical Outcomes Monitoring are useful for providing high-quality care to these patients. However, it is essential to integrate them with the patient's perspective according to Value-Based Healthcare paradigms. Patient-reported outcome measures (PROMs) and patient-reported experience measures (PREMs) are tools for assessing outcomes and experiences with health care from the patient's perspective. The aim of this paper is to collect and synthesize PROMs and main stakeholders' experience on the VC CP, according to a value-based approach.

**Materials and methods:**

To select the most appropriate instrument, a review was conducted on the main databases and official websites of specific institutions and organizations. In the second phase, a 2-round Delphi survey was conducted to assess the Reported Experience Measures (REMs) tool. Questions were evaluated according to four criteria (general relevance, evidence-based, measurability, actionability) and included if strong agreement was reached. A Principal Component Analysis (PCA) was executed. Cronbach's alpha and McDonald's omega were computed. Fisher's exact test and Wilcoxon rank sum test were used to compare ratings between groups. Descriptive statistics were performed for both PROMs and REMs instruments.

**Results:**

For PROMs assessment, EORTC QLQ-C30 questionnaire was selected and administered to 28 patients. Global Health Status/Quality of Life and Functional Scales Scores were high or very high, while symptoms scale reported low or medium scores. The final REMs consists of 22 questions for professionals and 16 for patients and caregivers. It was administered to 22 patients, 11 caregivers, 5 physicians, 2 nurses and 1 clinical senior manager. PCA identified 4 components. Scale reliability was acceptable (α = 0.75 95% CI: 0.61–0.85; ω = 0.69; 95% CI: 0.54, 0.82). A statistically significant difference between the patient/caregiver group and the professionals was found for items 8 (follow-up), 10 (perceived quality), 12 (safety), and 16 (climate) (*p* = 0.02; *p* = 0.03; *p* < 0.001; *p* < 0.001, respectively).

**Discussion:**

PROMs could provide new ways of intercepting patients' needs and feedback, thus acting on them. The proposed REMs tool would allow to detect information not available elsewhere, which, through Audit and feedback strategies, could lead to enhancement of healthcare experience, according to a value-based approach.

## 1. Introduction

Over the past few decades, neoplastic diseases have increased in incidence and prevalence, becoming one of the leading causes of death. However, some cancers are not very widespread. Vulvar cancer (VC) is one example. They account for about 5 percent of all cancers affecting the female genital tract (1). The annual incidence is 1–2/100,000 women. It is most frequently diagnosed in women aged 65–74 and accounts for <1% of cancers affecting the female gender (2, 3). Nevertheless, as the average life expectancy increases, cases of VC are likely to increase. To best deal with it, a drastic change in the organization of care pathways is required.

Patients with VC require multidisciplinary evaluation to design the best personalized clinical approach (4–6). This leads diverse health professionals to work and share their expertise and knowledge to create evidence-based decision-making according to the perspective of personalized medicine.

For these reasons, most hospitals have begun looking at new organizational paradigms to reshape hospital care delivery processes, moving away from the lines of traditional academic specialties and focusing primarily on patient needs (7). This is particularly true in the oncologic field where, in a Shared decision-making (SDM) context, patients and families are becoming more active, informed, and aware of the risks and benefits of various treatment options (8).

It involves the application of methods and tools to combine physician and manager perspectives to leverage the centrality of the person cared for, shifting from a “disease-centered” to a “person-centered” approach (9).

In 2022, Fondazione Policlinico Universitario A. Gemelli-IRCCS (FPG-IRCCS), a large tertiary care center located in Rome (Italy) and one of the largest Italian Oncological Centers, set up and implemented a VC critical pathway (CP). It encompasses the optimal care processes to improve quality and ensure that every care episode follows the most updated scientific evidence (10). In addition, consistent with the best updated scientific evidence, a multidisciplinary VC team was established in our institution. Structured around a core team and supplemented by a group of support specialists and a care manager, it is responsible for treatment strategies and individualized management. In their multidisciplinary tumor board meeting, about 260 cases are discussed annually (5). Finally, during the CP design phase, key performance indicators (KPIs) were selected and calculated to monitor the overall performance of the CP and ensure continuous improvement in the quality of care through audit & feedback (A&F) strategies.

However, an understanding of the experience and perspective of patients, caregivers and healthcare professionals involved in CP is missing in this context.

According to a value-based approach, this paper aims to collect and synthesize patient-reported outcomes and main stakeholders' experience on the CP for VC patients.

Specifically, the aim is to:

1) Collect and summarize the PROMs (Patient Reported Outcome Measures) of patients within the CP;
2) Measure, through validated questionnaires, the experience of various CP's stakeholders (patients, caregivers, physicians, nurses and managers), compare and assess the concordance of their perceptions regarding the issues of safety and quality of care.

## 2. Materials and methods

To develop the Gov → Value tool, a three-phase methodology was carried out: extensive literature review to identify a PROMs questionnaire for patients with VC; extensive literature review and Delphi validation of a Reported Experience Measures (REMs) questionnaire; pilot study.

### 2.1. Literature review concerning VC PROMs

In order to select a tool for measuring PROMs specifically in the case of patients with VC, validated in the Italian language, an extensive search of the main evidence-based and already validated questionnaires in the literature was carried out.

The main databases (PubMed, Scopus, Web of Science) and official websites of institutions and organizations with specific expertise in this field (AIOM, CIPOMO, EORTC, ICHOM, Istat) were consulted.

### 2.2. Literature review and Delphi validation of a REMs questionnaire

In the second stage, we scoured the scientific literature to identify relevant items for our REMs questionnaire. The main databases (PubMed, Scopus, Web of Science) were consulted. Based on the results of the review, we elaborated a set of items designed to assess experience as reported by various stakeholders (patients, caregivers, physicians, nurses, senior managers). A two-round Delphi survey was conducted to validate the final version of the REMs questionnaire. During the first round, a panel of experts was asked to express, for each question, their degree of agreement on a Likert scale of 1 to 3 (with 1 corresponding to the minimum-“Not Relevant” and 3 corresponding to the maximum-“Relevant”), based on the following four criteria:

- General Relevance.
- Support from scientific evidence.
- Measurability.
- Actionability.

The average of the four scores provided corresponded to the “overall” score, which was used to exclude items from the final set of indicators. In both rounds, the indicators with the lowest scores were excluded. The second round was also used to validate the final set of indicators.

The panel selection criteria for this study included at least one of the following: (i) publications on the topic of Clinical Governance; (ii) experience on the topic of Clinical Governance; (iii) knowledge and expertise of the phenomenon of Clinical Governance; and (iv) willingness and motivation to participate. All identified experts were contacted individually and asked for their willingness to participate in the Delphi process. Eleven experts, including healthcare managers, economists, and physicians, patient organization's representatives, were recruited. The team of experts was invited to complete the Delphi survey by email, through a Google Modules questionnaire. A cover letter explained the purpose, relevance, and usefulness of this survey. The answers were collected immediately and anonymously. At the end of the study and after the two Delphi Rounds, the instrument in its integrity (including all the questionnaires) was validated.

This methodology replicated one already applied by the team to another clinical setting (11).

The first Round of consultation started on the sixth of April 2022 and ended on the twentieth of April. The authors considered the following levels of agreement:

- “Strong Agreement”: “Overall” score of the item is equal to or more than 2.5 out of 3.0.
- “Agreement for Exclusion”: “Overall” score for each item is equal to or more than 2.0 out of 3.0.

In the presence of a “strong agreement for inclusion”, the indicator was included in the Second Round of the Survey. Items falling in the category “agreement for exclusion” were eliminated. The Second Round was structured as the First Round. For the final list of questions, the following levels of agreement were established:

- “Strong agreement for inclusion in the final list”: “Overall” score equal to or more than 2.5 out of 3.0.
- “Agreement for exclusion from final list”: mean of “Overall” score for each item <2.0 out of 3.0.

The Second Round of consultation started on the twenty-fifth of April 2022 and ended on the fifth of May 2022.

### 2.3. Pilot study

In the third stage, the final version of the Gov➔Value Tool (REMs questionnaire plus PROMs questionnaire) was tested in a sample of VC patients and their care team.

The pilot study was monocentric, taking place in the FPG-IRCCS VC outpatient setting, between May and June 2022.

Patients with the following ICD-9-CM codes: 184.4 (vulvar malignant tumor, unspecified), 196.5 (secondary and unspecified malignant tumors of the lymph nodes of the inguinal region and lower limb), 196.6 (secondary and unspecified malignant tumors of intrapelvic lymph nodes), 196.2 (secondary and unspecified malignant tumors of intra-abdominal lymph nodes), caregivers, nurses, physicians, and clinical managers were recruited from the VC CP of the FPG-IRCCS. Data regarding age, co-morbidities and demographics were collected face-to-face during clinical examinations performed by the care manager.

Given the pilot nature of our study, no standard sample sizing is necessary. Rules of practice for in-house pilot studies indicate 20 patients as the minimum sample (12).

#### 2.3.1. Inclusion criteria

All patients that are more than 18 years old and with malignant VC diagnosed on the VC CP are enrolled in the study and invited to reply to the questionnaire.

The eligible caregivers, instead, must be more than 18 years old and assist patients diagnosed with malignant VC included in the CP.

The eligible healthcare professionals (physician/nurse/manager) work in the Vulvar Pathology outpatient setting.

All the people included in the study must give the informed consent to the processing of data for research purposes.

#### 2.3.2. Exclusion criteria

Patients who are not able to understand the questions of the questionnaire (e.g., cognitive capabilities alteration, non-comprehension of Italian language) were excluded.

The caregivers excluded are those who are not able to understand the questions of the questionnaire.

Other patients and caregivers excluded are those with no informed consent to the processing of data for research purposes, as well.

#### 2.3.3. Survey administration

REMs and PROMs questionnaires were collected in a self-completed manner between May and June 2022. For patients unable to complete the questionnaire on their own, who expressed their willingness to participate in the study, completion support was offered, to ensure equity of participation.

#### 2.3.4. Processing of personal data

An informative note about the study was provided to respondents. Informed consent for the processing of data was required, complying with General Data Protection Regulation, 2018. In the module for informed consent collection, the freedom of withdrawing the consent in any moment is specified.

#### 2.3.5. Institutional review board approval

The research protocol was approved by the Ethics Committee of the Fondazione Policlinico Gemelli, Rome.

#### 2.3.6. Data analysis

##### 2.3.6.1. PROMs questionnaire

A descriptive analysis of the collected data was performed. The scores obtained from the PROMs questionnaires, linearly transformed on a scale from 0 to 100, were summarized and reprocessed using appropriate statistical methodologies, as indicated in EORTC QLQ-C30 manual (13). Mean, standard deviation (SD), median and interquartile range (IQR) were used for quantitative variables.

##### 2.3.6.2. REMs questionnaire

With regard to the REMs questionnaire, for categorical variables, absolute and relative frequency were provided, whereas mean, SD, median and IQR were used for quantitative variables. The Shapiro-Wilk test was applied to test the Gaussian distribution of the quantitative variables. A scale score was calculated by adding up individual items from the REMs questionnaire. A Principal Component Analysis (PCA) was run to collapse the questionnaire variables into a smaller number of principal components accounting for a large share of variance. The PCA was based on polychoric correlations, given the ordinal nature of data. A varimax rotation was applied, thus obtaining rotated components (RCs). Bartlett's test of sphericity and the Kaiser-Meyer-Olkin test were performed to check PCA's assumptions. Cronbach's alpha (>0.7 considered satisfactory) and McDonald's omega (>0.7 considered sufficient) were computed to assess questionnaire reliability. Missing data were handled through pairwise deletion, whenever possible. In order to compare ratings between groups, Fisher's exact test was used, based on indications on individual items' comparisons (14), while Wilcoxon rank sum test was used for the total scale. Effect size was reported, as well, by means of Cramer's V and r, reported with a 95% confidence interval. Effect size interpretation was based on commonly followed recommendations in published literature (15, 16).

Values of *p* < 0.05 were considered statistically significant. All statistical analyses were carried out in R software, version 4.2.0 (CRAN^®^, R Core 2022) within the RStudio platform, version 2022.02.3 + 492 (2009–2022 RStudio, PBC).

## 3. Results

### 3.1. Literature review

#### 3.1.1. PROMs

As a result of our literature review, EORTC QLQ-C30 questionnaire was selected (Appendix 1). It is already validated and consists of 30 areas comprising different scales, implemented to measure physical, psychological and social functions of cancer patients. The first 28 questions have four different answers: 1 = No; 2 = A little; 3 = A lot; 4 = Very Much.

The last two, instead, have a Likert scale from 1 to 7 as possible answers. The questionnaire is available at https://qol.eortc.org/questionnaire/eortc-qlq-c30/. Linear transformation was executed according to the dedicated manual (13) (Table 1).

**Table 1:** Results of EORTC QLQ-C30 questionnaire.

	**Num. of items**	**Item numbers**	**Raw Score**	**Range**	**Linear transformation**	**Linear transformation color interpretation**
**Global health status/QoL**						
Global health status/QoL	2	29, 30	4.63	6	60.51	
**Functional scales**						
Physical functioning	5	1 to 5	2.13	3	62.32	
Role functioning	2	6.7	2.41	3	52.9	
Emotional functioning	4	21 to 24	2.13	3	62.34	
Cognitive functioning	2	20.25	1.54	3	81.88	
Social functioning	2	26.27	1.72	3	76.09	
**Symptom scales/items**						
Fatigue	3	10,12,18	2.45	3	48.31	
Nausea and vomiting	2	14.15	1.46	3	15.22	
Pain	2	9.19	1.93	3	31.16	
Dyspnoea	1	8	1.57	3	18.84	
Insomnia	1	11	2.22	3	40.58	
Appetite loss	1	13	1.74	3	24.64	
Constipation	1	16	1.61	3	20.29	
Diarrhea	1	17	1.35	3	11.59	
Financial difficulties	1	28	1.48	3	15.94	

**0–30** corresponds to dark red. **31–50** corresponds to orange. **51–70** corresponds to light green. **71–100** corresponds to dark green.

#### 3.1.2. REMs

As a result of the literature review, a questionnaire was defined as follows:

o Doctor: 22 questions (14 Quality; 8 Safety);
o Nurse: 22 questions (14 Quality; 8 Safety);
o Senior Manager: 22 questions (14 Quality; 8 Safety);
o Patient: 16 Questions (10 Quality; 6 Safety);
o Care manager: 16 questions (10 quality; 6 safety);

Some questions are identical, while others were reformulated considering the user of the questionnaire. 18 questions were ranked through a 4-point Likert score:1 = no; 2 = a little; 3= rather much; 4 = very much. 4 questions (items 6, 9, 10, 20) were ranked on a dichotomic basis: yes; no.

### 3.2. Delphi validation of REMs questionnaire

#### 3.2.1. First round of consultation

Ten (91%) out of eleven experts recruited responded to the First Round.

The analytical results are reported by questions and by evaluation criterion (**Annex 1**).

All the sections (Physicians, Nurses, Senior Manager, Patients, Caregivers) were validated entirely in First Round and they were all included in the Second Round.

However, the “Nurses' quality Section” received the lowest scores on some items.

#### 3.2.2. Second round of consultation

Participation in the consultation was completed by 10 out of 10 participants (100%) and considered valid.

As well as in the first round of consultation, all the questionnaire sections received a positive evaluation from the experts and were validated entirely.

The average of each dimension of each perspective is 2.7 in the Second Round.

The resulting Five-sections questionnaire is composed of 72 questions, divided as follows (**Annex 1**):

1. Physicians: 22 questions (14 Quality Section; 8 Safety Section);
2. Nurses: 22 questions (14 Quality Section; 8 Safety Section);
3. Senior manager: 22 questions (14 Quality Section; 8 Safety Section);
4. Patients: 16 questions (10 Quality Section; 6 Safety Section);
5. Caregivers: 16 questions (10 Quality Section; 6 Safety Section).

### 3.3. Pilot study

#### 3.3.1. PROMs

Twenty-eight women with VC were identified during the study period, and response rate was 85.71% (*N* = 24). The median age was 64 (Interquartile Range = 22).

The majority of respondents were in post-treatment phase (follow-up), counting for the 95.83%.

The most frequent functional difficulties encountered by women mainly concerned their roles: they felt limited in their job (39.12%, *N* = 9 reported “a lot”/“very much” as level of limitation) and in their typical free-time activities (41.67%, *N* = 10 reported level “a lot”/“very much”). As concerns symptoms, difficulties in sleeping (39.12%%, *N* = 9 answered “a lot”/“very much”) and weakness were common (39.12%, *N* = 9 answered “a lot”/“very much”) and they frequently felt tired (41.67%, *N* = 10 answered “a lot”/“very much”).

On the status of their global health (QoL), on a range of 1/7, the women in a level ≥5 for “Health in the last 7 days” were 11 (45.83%). For an evaluation, instead, of their “Quality of life in the last 7 days” 10 women (41.67%) reported levels ≥5.

Table 1 reports results of PROMs administration.

#### 3.3.2. REMs' results

Twenty-two patients were included in the pilot study. All of the available caregivers (*n* = 11) accepted to respond. Physicians (*n* = 5), nurses (*n* = 2) and a clinical senior manager (*n* = 1), with experience in the treatment of women with VC, were also interviewed.

With regard to REMs questionnaire, mean, standard deviation (SD), median and interquartile range (IQR) of each Likert item's responses, classified into 2 categories, namely patients/caregivers and professionals, are displayed in Table 2. As to dichotomic items, Table 3 reports absolute and relative frequency.

**Table 2:** Mean, SD, median and IQR of responses to each Likert item, classified by respondent group (patient/caregiver or professional).

			**Mean (SD)**		**Median(IQR)**	
**Item no.**	**Subject**	**Missing (N)**	**Patient/caregiver**	**Professional**	**Patient/caregiver**	**Professional**
1	Care accessibility	0	3.52(0.87)	3.63(0.52)	4 (0.0)	4(0.25)
2	Punctuality	0	3.27 (0.88)	3 (0.76)	4 (1)	3 (0.5)
3	Information provided	0	3.7 (0.81)	3.5 (0.76)	4 (0)	4 (1)
4	Visit duration	0	3.61 (0.86)	3.38 (0.74)	4 (0)	3.5 (1)
5	Shared decision making	0	3.67 (0.82)	3.63 (0.74)	4 (0)	4 (0.25)
7	Informative material	0	2.64 (1.37)	1.63 (1.06)	3 (3)	3 (1)
8	Follow-up	0	3.97 (0.17)	3.63 (0.52)	4 (0)	4 (1)
11	Facility accessibility	0	3.06 (1.06)	2.5 (0.93)	3 (1)	2.5 (1)
12	Safety	0	3.76 (0.66)	3.25 (0.71)	4 (0)	3 (1)
13	Therapy risks/benefits	0	3.55 (0.79)	3.13 (0.83)	4 (1)	3 (1.25)
14	Adverse reaction	6	2.96 (1.26)	3.25 (0.89)	4 (2)	3.5 (1.25)
15	Polytherapy	6	2.93 (1.33)	3.38 (0.74)	4 (2.5)	3.5 (1)
16	Climate	0	3.73 (0.76)	3.13 (0.64)	4 (0)	3 (0.25)
17	Clinical outcome	0	–	2 (0.92)	–	2 (2)
18	Audit	0	–	2.88 (0.83)	–	3 (1.25)
19	Multidisciplinarity	0	–	3.13 (0.99)	–	3.5 (2)
21	Error reporting	0	–	3.13 (1.13)	–	3.5 (1.25)
22	Safety culture	0	–	2.88 (0.83)	–	3 (1.25)

**Table 3:** Percentage of responses to dichotomic items.

**Item n**.	**Missing (N)**	**Levels**	**Patients/caregiver (%)**	**Professionals (%)**
Item 6	2	No	1 (3.2%)	0 (0%)
				Yes	30 (96.8%)	8 (100%)
Item 9	2	No	15 (45.5%)	1 (16.7%)
				Yes	18 (54.5%)	5 (83.3%)
Item 10	1	No	25 (78.1%)	3 (37.5%)
				Yes	7 (21.9%)	5 (62.5%)
Item 20	0	No	–	3 (37.5%)
				Yes	–	5 (62.5%)

**Figure 1:**
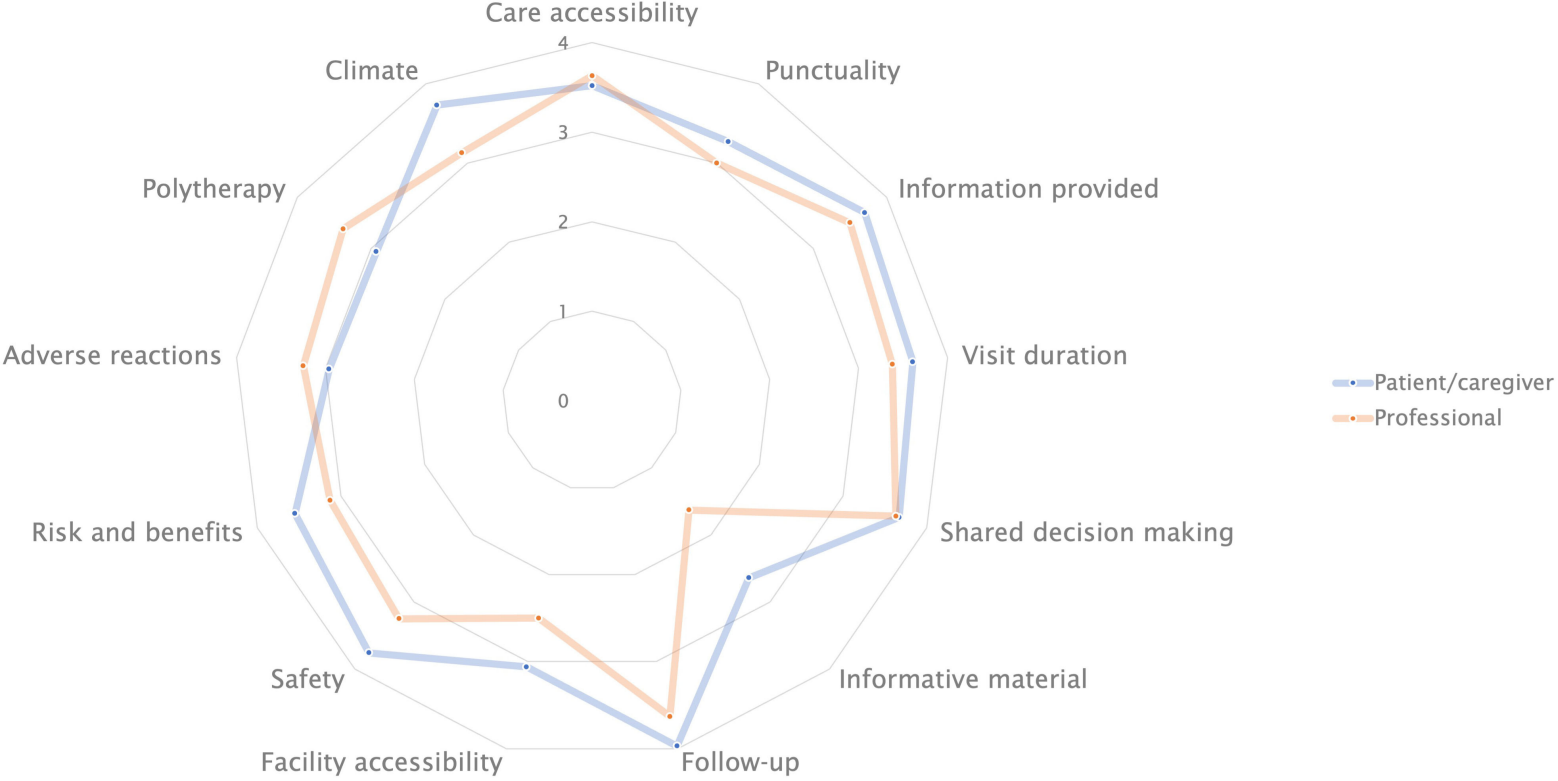
Radar plot showing mean scores for item topic, as assigned by professionals and patients/caregivers.

A radar plot summarizing mean responses provided by professionals and patients/caregivers is shown in Figure 1.

**Figure 2:**
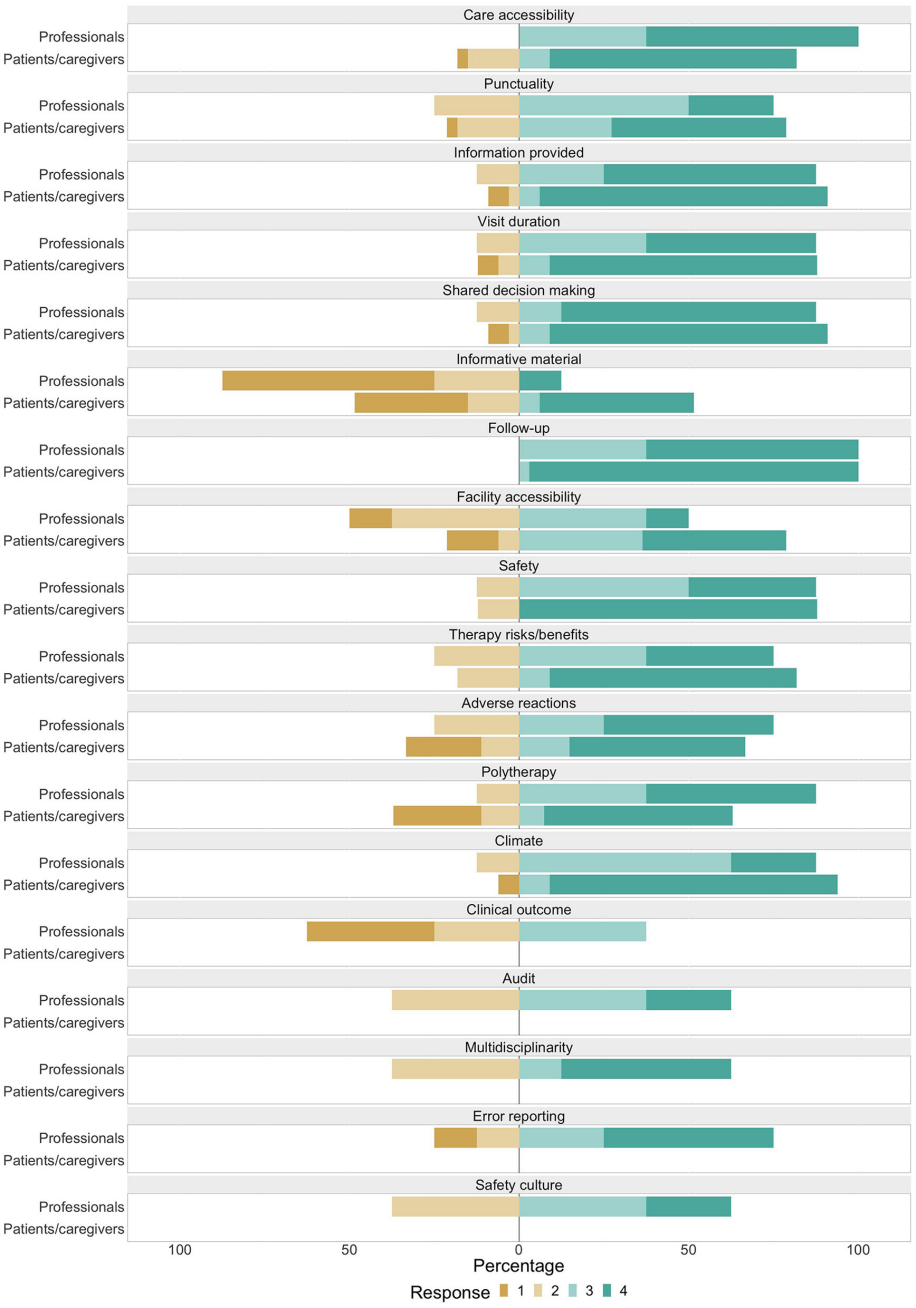
Barplot showing distribution of Likert items' responses among various scores, classified by group of raters. Scores 1, 2, 3, and 4, respectively correspond to responses: no, a little, rather much, very much.

A barplot showing ratings for each item, classified by group of raters (patients/caregivers and professionals) is depicted in Figure 2.

##### 3.3.2.1. PCA

The first 4 components accounted for 85% of variance in the dataset (Table 4). Explanation of therapy risks, visit duration, information provided, safety and shared decision-making mainly contribute to RC 1. RC 2 mainly consists of the following variables: punctuality, patient-physician climate and accessibility in terms of ease of visit reservation. Clear description of adverse reactions and polytherapy risks largely contribute to RC 3. Provision of informative material, indications on follow-up steps and facility accessibility have the highest loadings on RC 4. RC 1 can be interpreted as general care quality features of the visit itself. RC 3 is mainly concerned with the experience related to medical therapy prescription. RC 2 consists of aspects related to the visit experience, such as punctuality and physician-patient climate. Lastly, RC 4 mostly has to do with follow-up relevant activities.

**Table 4:** Item loadings on RCs and cumulative percentage of variance accounted for by RCs.

	**RC1**	**RC3**	**RC2**	**RC4**
Therapy risks/benefits	**0.87**	0.05	0.34	0.18
Visit duration	**0.86**	0.28	0.30	−0.08
Information provided	**0.83**	0.40	−0.23	0.11
Safety	**0.79**	−0.02	0.06	0.11
Shared decision making	**0.73**	0.45	−0.40	0.00
Adverse reactions	0.36	**0.86**	0.23	0.04
Polytherapy	0.37	**0.82**	−0.03	0.24
Climate	0.31	−0.47	**0.72**	0.03
Care accessibility	−0.25	0.35	**0.84**	0.10
Punctuality	0.23	−0.01	**0.67**	0.27
Informative material	−0.14	0.18	0.15	**0.92**
Follow-up	0.46	0.03	0.12	**0.84**
Facility accessibility	0.09	**−0.64**	0.16	**0.62**
Cumulative % of variance explained by RCs	31	52	69	85

Loadings > | 0.6 | are highlighted in bold.

##### 3.3.2.2. Reliability analysis

When considering 13 4-point Likert items administered to all respondents (patients, caregivers and professionals), Cronbach's alpha was satisfactory (α = 0.75; 95% CI: 0.61–0.85). McDonald's Omega was similarly sufficient (ω = 0.69; 95% CI: 0.54, 0.82).

Cronbach's alpha if an item is deleted ranged from 0.48 to 0.77. Omega if an item is deleted ranged from 0.54 to 0.75. Specifically, item 7 and item 11 would increase omega notably.

When stratified by RC, Cronbach's alpha was 0.87 (95% CI: 0.80–0.92) in RC1, 0.91 (95% CI: 0.83–0.96) in RC3, 0.6 (95% CI: 0.38–0.81) in RC2 and 0.49 (95% CI: 0.14–0.71) in RC4.

When applied to the 13 4-point Likert items administered to patients and caregivers, Cronbach's alpha was satisfactory (α = 0.73; 95% CI: 0.56–0.85), while McDonald's Omega was nearly sufficient (ω = 0.64; 95% CI: 0.43, 0.80). If an item is dropped, alpha ranged from 0.37 to 0.76, while Omega was in the range from 0.32 to 0.72. Still, items 7 and 11 were negatively affecting omega the most.

Considering the 18 4-point Likert items for the professionals' group, Cronbach's alpha was fairly high (α = 0.84; 95% CI: 0.62–0.96) and McDonald Omega was sufficient (ω = 0.82; 95% CI: 0.58−0.96). Alpha ranged from 0.82 to 0.86 and omega from 0.32 to 0.87, in the case of removing an item. Deletion of items 2 and 21 would increase omega the most.

##### 3.3.2.3. Comparison between scores from patients/caregivers and professionals

Results from comparison between professionals' and patients/caregivers' responses to each item are reported in Table 5.

**Table 5:** Results of comparison between responses to each item from the professionals' and patients/caregivers' groups.

**Item**	**Topic**	***p*** **-value**	**Effect size (95% CI)**	**Effect size magnitude**
Item1	Accessibility	0.23	0.35 (0.05–0.6)	Large
Item2	Punctuality	0.51	0.24 (0–0.51)	Medium
Item3	Information provided	0.16	0.33 (0.03–0.58)	Large
Item4	Visit duration	0.12	0.35 (0.06–0.6)	Large
Item5	Shared decision making	0.68	0.21 (0–0.49)	Medium
Item6	Care plan	1	0.08 (0–0.38)	–
Item7	Informative material	0.26	0.31 (0.01–0.57)	Large
Item8	Follow-up	**0.02**	0.46 (0.18–0.68)	Medium
Item9	Care pathway	0.43	0.16 (0–0.45)	Small
Item10	Perceived quality	**0.03**	0.36 (0.06–0.6)	Medium
Item11	Facility accessibility	0.09	0.41 (0.12–0.64)	Large
Item12	Safety	**< 0.001**	0.67 (0.47–0.82)	Large
Item13	Therapy risks/benefits	0.08	0.35 (0.048–0.59)	Large
Item14	Adverse reaction	0.36	0.29 (0–0.57)	Medium
Item15	Polytherapy	0.09	0.42 (0.1–0.66)	Large
Item16	Climate	**< 0.001**	0.65 (0.43–0.8)	Large
Scale		0.25	0.2 (0.01−0.48)	Small

P-value, Cramer's V with 95% confidence interval and interpretation are provided.

A statistically significant difference between the patient/caregiver group and the professionals was found for items 8, 10, 12 and 16 (*p* = 0.02; *p* = 0.03; *p* < 0.001; *p* < 0.001, respectively). Effect size was medium for item 8 and 10 and large for items 12 and 16.

Items 1, 2, 3, 4, 5, 6, 7, 9, 11, 13, 14, 15 and the total scale show no statistically significant difference, when comparing responses from patients/caregivers with those from professionals.

A comparison between scores from each individual group of stakeholders (physicians, nurses, manager, patients, caregivers) led to statistically significant differences for items 8 (*p* = 0.002), 12 (*p* = 0.003) and 16 (*p* < 0.001).

## 4. Discussion

The main goal of our research was to collect and synthesize patients' and key healthcare stakeholders' experience on a CP dedicated to women with VC, as only a few tools have been developed in oncological care to explore these items (17).

In several countries, patient-reported outcome measures (PROMs) and patient-reported experience measures (PREMs) are widely used in research and performance evaluation (18). This item is crucial as healthcare organizations could use PREMs and PROMs to: (a) improve the quality of care on an individual level, *via* a patient-centered approach through the implementation of personalized care, notably due to consideration of patients' concerns and needs; (b) improve diagnosis of diseases and potentially reduce their severity, *via* more regular or systematic assessment of the effectiveness of care and monitoring of disease progression; (c) increase patient information, communication and shared medical decision-making (19), thus paving the way for precision and personalized medicine (20, 21).

The present work aims to move beyond the mere patient experience (PREM) assessment by including all the key CP stakeholders' viewpoint. By doing so, PREMs were integrated with doctors, nurses and clinical leaders and caregivers' perspective, thus becoming “Reported Experience Measures” (REMs).

As no validated questionnaires on REMs were available, a specific one was designed, constructed, validated through the Delphi methodology and administered.

The proposed tool aims to analyze the same phenomenon (comparable dimensions of quality and safety and all referred to the same care event) from different perspectives.

Such an approach would recall the application of lean tools to improve quality and safety of care in testing or diagnostics (22) by comparing quality and safety experiences based on five different perspectives. It would be a development of the “Go to the Gemba”, the Japanese word meaning “go to the place” through the processes of care (23), so as to implement quality improvement initiatives where concordance among the different perspectives lacks.

To this end, it becomes essential to find organizational solutions that combine a high degree of specialization, technical and scientific advances, multidisciplinary and multiprofessional coordination, and patient participation (24).

To pursue such an approach, an internal organization consistency is required: (a) first, a standardized CP has to be developed by a dedicated multidisciplinary team to provide “case by case”, high-quality diagnosis, and evidence-based decision-making in the context of personalized medicine; (b) secondly, a set of KPIs has to be defined by the hospital monitoring system. This, combined with an A&F system, creates a virtuous environment with the primary goal of improving health provider performance and healthcare outcomes.

To our knowledge, this is among the first few studies assessing the feasibility of collecting data on different stakeholders' experience at a given “point of care” event, within the patient CP, so as to assess the different perspectives on crucial dimensions of quality and safety and elicit information to improve oncological care.

Such requirements matched with the management of vulvar cancer, where, unlike other oncology conditions, such as Breast Cancer (25, 26), the patient's experience has been less studied.

Alimena et al. (27), who examined PROMs in a typical clinic population of vulvar cancer patients, administered the following validated tools: the European Organization for the Research and Treatment of Cancer Quality of life Questionnaire (EORTC QLQ-C30), the Patient Reported Outcome Measurement Information System (PROMIS) Emotional and Instrumental Support Questionnaires, and the Functional Assessment of Cancer Therapy-Vulvar (FACT-V) questionnaire.

In our study, as the other PRO questionnaires were not validated in Italian, we were able to use only the EORTC QLQ-C30 questionnaire, and as to REMs questionnaire, the one we defined was tested.

Our study has a number of strengths: it makes use of an innovative tool, in line with recent literature developments; it deploys a rigorous methodological approach throughout all of the study phases; it originally provides various perspectives on health care experience.

Our study, however, does not come without its limitations. First, by default, all studies on vulvar cancer patients have relatively small populations. In addition, as regards the PROMs questionnaire administration, differently from a previous study (27), in our study it was not possible to use disease-specific questionnaires because the Italian version was not available (e.g., PROMIS and FACT-V). As a consequence, we chose to use the European Organization for the Research and Treatment of Cancer Quality of life Questionnaire (EORTC QLQ-C30). It is a frequently used patient-reported outcome instrument to assess health-related quality of life of patients with cancer. However, it is not designed to stratify by comorbidities.

Further limitations include the fact that, in questionnaire administration, a paper version was preferred. Paper questionnaires involve several time-consuming and costly steps. However, this choice is mainly related to the mean age of the patients that distinguish our cohort. Indeed, older populations typically suffer from low digital literacy, even though the proportion of elderly using digital technology has increased exponentially (28, 29).

The Delphi methodology was used to validate the REMs questionnaire. This methodology is used to combine expert knowledge and opinion to arrive at an informed group consensus on a complex problem. However, some methodological limitations should be taken into account (such as starting with provided material and questions may not be representative, the process tends to eliminate extreme positions and force a middle-of-the-road consensus and is also vulnerable to high dropout rates due to the large time commitment required) (30).

Both PCA and reliability analysis would benefit from a larger sample (31, 32). Additionally, we used Cronbach's alpha, which is a widely used coefficient for reliability assessment, despite a wide range of limits, largely based on hard-to-meet assumptions. We still reported it in light of its popularity, but decided to complement it with McDonald's Omega, which is recognized as more accurate (33). When comparing ratings, we considered patients and caregivers as one group and professionals as another, in order to avoid a fragmentation of our small sample, while still maintaining a focus on the main perspectives at stake. Furthermore, ideally, we should collect data on each individual care episode from the various stakeholders involved. This would allow a more comprehensive and specific interpretation of data.

In addition, we are aware that our search focused on patient experiences in the outpatient care setting within the hospital, and not on the whole pathway, as needed in cancer care.

In terms of future perspectives, longitudinally-administered PROMs questionnaires allow clinicians to keep track of clinical outcomes as reported by patients, as a valuable addition to clinical performance monitoring systems. This tool could provide new ways of intercepting patients' needs and feedback, thus acting on them. With regard to REMs, A&F practice based on results from the proposed questionnaire might play a key role in improving professional practice. Thus, our tool would allow to detect information not available elsewhere, with potential enhancement of healthcare experience for both patients and professionals, according to a value-based approach.

Our team emphasize how Patient-reported outcome measures (PROMs) and patient-reported experience measures (PREMs) are complementary and necessary tools to improve quality and safety.

Depending on the scores reported from individual perspectives, *ad hoc* improvement actions, such as A&F interventions, will be taken if discrepancies or critical issues emerge. If designed optimally and used in the right context, A&F can play an important role in improving professional practice.

This is especially true in the field of oncology, where it increasingly plays a leading role through the creation of SDM processes (34).

## Data availability statement

The raw data supporting the conclusions of this article will be made available by the authors, without undue reservation.

## Ethics statement

The studies involving human participants were reviewed and approved by Ethics Committee of Fondazione Policlinico Universitario A. Gemelli IRCCS. The patients/participants provided their written informed consent to participate in this study.

## Author contributions

EM and CA: conceptualization, methodology, data processing, writing of original draft, and project management. AP: methodology, data processing, formal analysis, and writing of original draft. AB: conceptualization, review, editing of manuscript, and supervision. SF: data collection, review, editing of manuscript, and supervision. LG: recruitment of participants and data collection. GS, GG, and LT: review and supervision. All authors approved the contributions, read, and approved the final manuscript.
