# Supplementary material for: Gov➔Value: How to combine reported quality experiences and patient-reported outcome measures. First results on vulvar cancer patients in an Italian Research Hospital
Source: Front Public Health. 2022 Dec 20;10:1014651. doi: 10.3389/fpubh.2022.1014651 (PMC9807601; doi:10.3389/fpubh.2022.1014651)
Supplement: Supplementary file 1 [file Data_Sheet_1.pdf]

REMs questionnaire and Delphi results

Summary tables below: in green, the indicators that reached positive evaluations for inclusion with “strong agreement”; yellow represents “agreement for exclusion” for the items with a score equal to or more than 2.0 out of 3.0. No indicator reached a score lower than 2.0 for the exclusion from the final list.

PERSPECTIVE: PHYSICIAN			DELPHI: FIRST ROUND				OVERALL	DELPHI: SECOND ROUND				OVERALL
SECTION: QUALITY			General relevance	Support from scientific evidence	Measurability	Actionability		General relevance	Support from scientific evidence	Measurability	Actionability	
	SCORE:	SOURCE:										
Does the patient usually complain about the difficulty of making an appointment?	no<br>a little<br>rather much<br>very much	Zhao P, Yoo I, Lavoie J, Lavoie BJ, Simoes E. Web-Based Medical Appointment Systems: A Systematic Review. J Med Internet Res. 2017;19(4):e134. Published 2017 Apr 26. doi:10.2196/jmir.6747	3.0	3.0	2.4	2.2	2.7	3.0	2.9	2.7	2.9	2.9
Were you able to respect the visiting hours?	no<br>a little<br>rather much<br>very much	Hyo Jung Tak, Gavin W. Hougham, Atsuko Ruhnke, Gregory W. Ruhnke. The effect of in-office waiting time on physician visit frequency among working-age adults, 2014	2.5	2.5	2.6	2.6	2.6	2.6	2.4	2.5	2.6	2.5
Were you able to inform the patient about the Diagnosis/Therapy/Follow-up?	no<br>a little<br>rather much<br>very much	https://www.ahrq.gov/sites/default/files/wysiwyg/cahps/surveys-guidance/cg/cg-comparison-3-4.pdf	2.8	2.9	2.6	2.4	2.7	3.0	2.9	2.4	2.3	2.6
According to the time allocated, were you able to respond comprehensively to the patient's and/or caregiver's requests?	no<br>a little<br>rather much<br>very much	https://www.ahrq.gov/sites/default/files/wysiwyg/cahps/surveys-guidance/cg/cg-comparison-3-4.pdf	2.9	2.7	2.8	2.7	2.8	2.8	2.8	2.8	2.7	2.8
According to the time allocated, did you share the care plan with the patient?	no<br>a little<br>rather much<br>very much	Canberra Health Services. The Foundation for Exceptional Care Clinical Governance Framework 2020–2023	2.4	2.6	2.4	2.4	2.5	2.6	2.4	2.4	2.8	2.6
If yes, did it leave a trace on the Company Information System?	yes<br>no		2.7	2.5	2.6	2.5	2.6	2.7	2.6	2.6	2.7	2.7
During the visit, were you able to provide information material on the disease and/or voluntary associations?	no<br>a little<br>rather much<br>very much		2.6	2.5	2.7	2.7	2.6	2.7	2.3	2.4	2.4	2.5
Depending on the time spent, were you able to include information for subsequent appointments (time, place and contact details) in the documentation issued to the patient?	no<br>a little<br>rather much<br>very much	Care Quality Commission. NHS Core Inpatient Survey 2021. Survey handbook. Ipsos MORI, Coordination Centre for Mixed Methods. Version 1.	3.0	3.0	2.4	2.2	2.7	2.9	2.8	2.6	2.5	2.7
Is there a Clinical Pathway (CP) relating to the patient's health problem?	yes<br>no	Edward Abrahams, Alan Balch, Patricia Goldsmith, Marcia Kean, Amy M. Miller, Gilbert Omenn, Ellen Sonet, John Sprandio, Courtney Tyne, Kimberly Westrich; Clinical Pathways: Recommendations for Putting Patients at the Center of Value-Based Care. Clin Cancer Res 15 August 2017; 23 (16): 4545–4549. https://doi.org/10.1158/1078-0432.CCR-17-1609	3.0	3.0	3.0	3.0	3.0	3.0	3.0	3.0	3.0	3.0
Do you systematically receive clinical performance data on the health problem from the management?	no<br>a little<br>rather much<br>very much	Specchia, M.L., La Torre, G., Siliquini, R. et al. OPTIGOV - A new methodology for evaluating Clinical Governance implementation by health providers. BMC Health Serv Res 10, 174 (2010).	2.8	2.9	2.4	2.6	2.7	3.0	2.9	2.7	2.6	2.8
Together with the care team, do you have the opportunity to discuss clinical performance data and/or quality of care? (audits, M&M meetings etc.)	no<br>a little<br>rather much<br>very much	Specchia, M.L., La Torre, G., Siliquini, R. et al. OPTIGOV - A new methodology for evaluating Clinical Governance implementation by health providers. BMC Health Serv Res 10, 174 (2010).	2.8	2.5	2.7	2.6	2.7	2.7	2.5	2.6	2.5	2.6
Is there dedicated time to discuss the patient's health problem in a multidisciplinary way?	no<br>a little<br>rather much<br>very much	Specchia, M.L., La Torre, G., Siliquini, R. et al. OPTIGOV - A new methodology for evaluating Clinical Governance implementation by health providers. BMC Health Serv Res 10, 174 (2010).	3.0	2.9	2.9	3.0	2.9	2.9	2.9	3.0	3.0	3.0
Is there any time planned for refresher/training/empowerment activities addressed to you in relation to this health problem?	yes<br>no	Specchia, M.L., La Torre, G., Siliquini, R. et al. OPTIGOV - A new methodology for evaluating Clinical Governance implementation by health providers. BMC Health Serv Res 10, 174 (2010).	2.8	2.6	2.7	2.7	2.7	2.7	2.6	2.8	2.7	2.7
Does your company use questionnaires to assess organisational well-being/work experience?	yes<br>no	Al-Abri R, Al-Balushi A. Patient satisfaction survey as a tool towards quality improvement. Oman Med J. 2014;29(1):3-7. doi:10.5001/omj.2014.02	2.8	2.8	2.9	2.7	2.8	2.9	2.8	2.8	2.9	2.9
SECTION: SAFETY			General relevance	Support from scientific evidence	Measurability	Actionability	OVERALL	General relevance	Support from scientific evidence	Measurability	Actionability	OVERALL
	SCORE:	SOURCE:										

Have you been enabled to carry out your daily activities easily in relation to the patients' care needs (parking, architectural barriers, signposting, information box, waiting room seating)?	no<br>a little<br>rather much<br>very much	Matin BK, Williamson HJ, Karyani AK, Rezaei S, Soofi M, Soltani S. Barriers in access to healthcare for women with disabilities: a systematic review in qualitative studies. BMC Womens Health. 2021;21(1):44. Published 2021 Jan 30. doi:10.1186/s12905-021-01189-5	2.9	2.7	2.6	2.6	2.7	2.9	2.6	2.6	2.7	2.7
Do you feel that all conditions for the safe provision of services (physical, biological, environmental risks) e.g. needles, ICA, catheter-operated hand washing, fall risk, use of PPE have been made available to you?	no<br>a little<br>rather much<br>very much	Canberra Health Services. The Foundation for Exceptional Care Clinical Governance Framework 2020–2023	3.0	2.8	2.7	2.7	2.8	3	2.9	2.9	2.9	2.9
Did you have sufficient time to communicate risks and/or complications of the medical/surgical treatment to the patient?	no<br>a little<br>rather much<br>very much	Canberra Health Services. The Foundation for Exceptional Care Clinical Governance Framework 2020–2023	3	2.9	2.8	2.9	2.9	3	2.9	2.8	2.9	2.9
Did you have sufficient time to communicate possible adverse reactions of prescribed drugs to the patient?	no<br>a little<br>rather much<br>very much	Bomhof-Roordink H, Gärtner FR, van Duijn-Bakker N, van der Weijden T, Stiggelbout AM, Pieterse AH. Measuring shared decision making in oncology: Development and first testing of the iSHAREpatient and iSHAREphysician questionnaires. Health Expect. 2020 Apr;23(2):496-508. doi: 10.1111/hex.13015. Epub 2020 Feb 5. PMID: 32022350; PMCID: PMC7104639.	3	2.8	2.9	2.8	2.9	3	2.7	2.9	2.8	2.9
In the case of polypharmacotherapy, were the risks and/or complications of this treatment communicated and understood?	no<br>a little<br>rather much<br>very much	Robyn Clay-Williams et al. clinician safety culture and leadership questionnaire: refinement and validation in Australian public hospitals | International Journal for Quality in Health Care.<br>Anthony R. Cox and Tehreem F. Butt. Adverse Drug Reactions When the Risk Becomes a Reality for Patients.	2.8	2.7	2.5	2.6	2.7	2.7	2.7	2.6	2.6	2.7
Did your company provide you with a system for reporting possible errors?	no<br>a little<br>rather much<br>very much	Laura L. Gleeson et al. Safety culture in a major accredited Irish university teaching hospital: a mixed methods study using the safety attitudes questionnaire.<br>Canberra Health Services. The Foundation for Exceptional Care Clinical Governance Framework 2020–2023	2.7	2.6	2.5	2.5	2.6	2.8	2.6	2.6	2.5	2.6
Do you feel that the company concretely pursues a culture of learning from errors (training, audits, reporting)?	no<br>a little<br>rather much<br>very much	Specchia, M.L., La Torre, G., Siliquini, R. et al. OPTIGOV - A new methodology for evaluating Clinical Governance implementation by health providers. BMC Health Serv Res 10, 174 (2010).	2.8	2.8	2.6	2.6	2.7	2.6	2.6	2.6	2.6	2.6
Did you perceive a climate of hostility and/or aggressiveness on the part of patients during the service?	no<br>a little<br>rather much<br>very much		2.8	2.4	2.8	2.8	2.7	2.9	2.4	3.0	2.6	2.7

PERSPECTIVE: NURSE			DELPHI: FIRST ROUND				OVERALL	DELPHI: SECOND ROUND				OVERALL
SECTION: QUALITY			General relevance	Support from scientific evidence	Measurability	Actionability		General relevance	Support from scientific evidence	Measurability	Actionability	
	SCORE:	SOURCE:										
Does the patient usually complain about the difficulty of making an appointment?	no<br>a little<br>rather much<br>very much	Zhao P, Yoo I, Lavoie J, Lavoie BJ, Simoes E. Web-Based Medical Appointment Systems: A Systematic Review. J Med Internet Res. 2017;19(4):e134. Published 2017 Apr 26. doi:10.2196/jmir.6747	3.0	3.0	2.4	2.2	2.7	3.0	2.9	2.7	2.9	2.9
Were you able to respect the visiting hours?	no<br>a little<br>rather much<br>very much	Hyo Jung Tak, Gavin W. Hougham, Atsuko Ruhnke, Gregory W. Ruhnke. The effect of in-office waiting time on physician visit frequency among working-age adults, 2014	2.4	2.6	2.4	2.4	2.5	2.6	2.4	2.4	2.4	2.5
Depending on the time allocated, was the patient informed about the Diagnosis/Therapy/Follow-up?	no<br>a little<br>rather much<br>very much	https://www.ahrq.gov/sites/default/files/wysiwyg/cahps/surveys-guidance/cg/cg-comparison-3-4.pdf	2.8	2.9	2.6	2.4	2.7	3.0	2.9	2.4	2.3	2.6
Depending on the time allocated, were you able to respond comprehensively to the patient's and/or caregiver's requests?	no<br>a little<br>rather much<br>very much	https://www.ahrq.gov/sites/default/files/wysiwyg/cahps/surveys-guidance/cg/cg-comparison-3-4.pdf	2.8	2.8	2.2	3.0	2.7	2.8	2.8	2.2	3.0	2.7
Depending on the time allocated, was the care plan shared with the patient?	no<br>a little<br>rather much<br>very much	Canberra Health Services. The Foundation for Exceptional Care Clinical Governance Framework 2020–2023	2.6	2.4	2.4	2.8	2.6	2.8	2.8	2.2	3.0	2.7
If yes, was a record left on the Company Information System?	yes<br>no		2.9	2.9	2.7	2.6	2.8	2.9	2.9	2.7	2.6	2.8
During the visit, was information material on the disease and/or voluntary associations provided?	no<br>a little<br>rather much<br>very much		2.6	2.2	2.2	2.4	2.5	2.7	2.3	2.4	2.4	2.5
Depending on the time dedicated, was information for subsequent appointments (time, place and contact details) included in the documentation issued to the patient?	no<br>a little<br>rather much<br>very much	Care Quality Commission. NHS Core Inpatient Survey 2021. Survey handbook. Ipsos MORI, Coordination Centre for Mixed Methods. Version 1.	3.0	3.0	2.4	2.2	2.7	2.8	3.0	2.6	2.6	2.7
Is there a Clinical Pathway (CP) related to the patient's health problem?	yes<br>no	Edward Abrahams, Alan Balch, Patricia Goldsmith, Marcia Kean, Amy M. Miller, Gilbert Omenn, Ellen Sonet, John Sprandio, Courtney Tyne, Kimberly Westrich; Clinical Pathways: Recommendations for Putting Patients at the Center of Value-Based Care. Clin Cancer Res 15 August 2017; 23 (16): 4545–4549. https://doi.org/10.1158/1078-0432.CCR-17-1609	3.0	3.0	3.0	3.0	3.0	3.0	3.0	3.0	3.0	3.0
Do you systematically receive clinical performance data on the health problem from the management?	no<br>a little<br>rather much<br>very much	Specchia, M.L., La Torre, G., Siliquini, R. et al. OPTIGOV - A new methodology for evaluating Clinical Governance implementation by health providers. BMC Health Serv Res 10, 174 (2010).	2.8	2.9	2.4	2.6	2.7	3.0	2.9	2.7	2.6	2.8
Together with the care team, do you have the opportunity to discuss clinical performance data and/or quality of care? (audits, M&M meetings etc.)	no<br>a little<br>rather much<br>very much	Specchia, M.L., La Torre, G., Siliquini, R. et al. OPTIGOV - A new methodology for evaluating Clinical Governance implementation by health providers. BMC Health Serv Res 10, 174 (2010).	2.8	3.0	2.6	2.6	2.7	3.0	3.0	2.6	2.6	2.8
Is time set aside for multidisciplinary discussion of the patient's health problem?	no<br>a little<br>rather much<br>very much	Specchia, M.L., La Torre, G., Siliquini, R. et al. OPTIGOV - A new methodology for evaluating Clinical Governance implementation by health providers. BMC Health Serv Res 10, 174 (2010).	2.8	2.6	2.6	2.6	2.7	2.8	2.8	2.2	3.0	2.7
Is there any time planned for refresher/training/empowerment activities addressed to you in relation to this health problem?	yes<br>no	Specchia, M.L., La Torre, G., Siliquini, R. et al. OPTIGOV - A new methodology for evaluating Clinical Governance implementation by health providers. BMC Health Serv Res 10, 174 (2010).	2.8	2.9	2.6	2.4	2.7	3.0	2.9	2.4	2.3	2.6
Does your company use questionnaires to assess organisational well-being/work experience?	yes<br>no	Al-Abri R, Al-Balushi A. Patient satisfaction survey as a tool towards quality improvement. Oman Med J. 2014;29(1):3-7. doi:10.5001/omj.2014.02	2.8	3.0	2.6	2.6	2.7	2.9	2.9	2.7	2.6	2.8
SECTION: SAFETY			General relevance	Support from scientific evidence	Measurability	Actionability	OVERALL	General relevance	Support from scientific evidence	Measurability	Actionability	OVERALL
	SCORE:	SOURCE:										
Have you been enabled to carry out your daily activities easily in relation to the patients' care needs (parking, architectural barriers, signposting, information box, waiting room seating)?	no<br>a little<br>rather much<br>very much	Matin BK, Williamson HJ, Karyani AK, Rezaei S, Soofi M, Soltani S. Barriers in access to healthcare for women with disabilities: a systematic review in qualitative studies. BMC Womens Health. 2021;21(1):44. Published 2021 Jan 30. doi:10.1186/s12905-021-01189-5	2.9	2.7	2.6	2.6	2.7	2.9	2.6	2.6	2.7	2.7
Do you feel that all conditions for the safe provision of services (physical, biological, environmental risks) e.g. needles, ICA, catheter-operated hand washing, fall risk, use of PPE have been made available to you?	no<br>a little<br>rather much<br>very much	Canberra Health Services. The Foundation for Exceptional Care Clinical Governance Framework 2020–2023	2.8	3.0	2.6	2.6	2.7	2.9	3.0	2.4	2.6	2.7
Did you have sufficient time to communicate risks and/or complications of the medical/surgical treatment to the patient?	no<br>a little<br>rather much<br>very much	Canberra Health Services. The Foundation for Exceptional Care Clinical Governance Framework 2020–2023	2.9	2.9	2.7	2.6	2.8	2.9	2.9	2.7	2.6	2.8

Did you have sufficient time to communicate possible adverse reactions of prescribed drugs to the patient?	no<br>a little<br>rather much<br>very much	Bomhof-Roordink H, Gärtner FR, van Duijn-Bakker N, van der Weijden T, Stiggelbout AM, Pieterse AH. Measuring shared decision making in oncology: Development and first testing of the iSHAREpatient and iSHAREphysician questionnaires. Health Expect. 2020 Apr;23(2):496-508. doi: 10.1111/hex.13015. Epub 2020 Feb 5. PMID: 32022350; PMCID: PMC7104639.	2.6	3.0	2.8	2.8	2.8	2.7	2.9	2.8	2.8	2.8
In the case of polypharmacotherapy, were the risks and/or complications of this treatment communicated and understood?	no<br>a little<br>rather much<br>very much	Robyn Clay-Williams et al. clinician safety culture and leadership questionnaire: refinement and validation in Australian public hospitals | International Journal for Quality in Health Care.<br>Anthony R. Cox and Tehreem F. Butt. Adverse Drug Reactions When the Risk Becomes a Reality for Patients.	2.9	2.7	2.2	2.3	2.5	2.9	2.9	2.3	2.4	2.6
Did your company provide you with a system for reporting possible errors?	no<br>a little<br>rather much<br>very much	Laura L. Gleeson et al. Safety culture in a major accredited Irish university teaching hospital: a mixed methods study using the safety attitudes questionnaire.<br>Canberra Health Services. The Foundation for Exceptional Care Clinical Governance Framework 2020–2023	2.8	2.8	2.2	3.0	2.7	2.7	2.9	2.4	2.8	2.7
Do you feel that the company concretely pursues a culture of learning from errors (training, audits, reporting)?	no<br>a little<br>rather much<br>very much	Specchia, M.L., La Torre, G., Siliquini, R. et al. OPTIGOV - A new methodology for evaluating Clinical Governance implementation by health providers. BMC Health Serv Res 10, 174 (2010).	3.0	2.2	3.0	2.4	2.7	2.6	2.8	2.6	2.4	2.6
Did you perceive a climate of hostility and/or aggressiveness on the part of patients during the service?	no<br>a little<br>rather much<br>very much		2.8	2.4	2.8	2.8	2.7	2.9	2.4	3.0	2.6	2.7

PERSPECTIVE: SENIOR MANAGER			DELPHI: FIRST ROUND				OVERALL	DELPHI: SECOND ROUND				OVERALL
SECTION: QUALITY			General relevance	Support from scientific evidence	Measurability	Actionability		General relevance	Support from scientific evidence	Measurability	Actionability	
	SCORE:	SOURCE:										
Do you receive complaints about the difficulty of making appointments?	no<br>a little<br>rather much<br>very much	Zhao P, Yoo I, Lavoie J, Lavoie BJ, Simoes E. Web-Based Medical Appointment Systems: A Systematic Review. J Med Internet Res. 2017;19(4):e134. Published 2017 Apr 26. doi:10.2196/jmir.6747	2.8	2.8	2.7	2.8	2.8	2.9	2.9	2.7	2.9	2.8
Has the management put you in a position to enforce visiting hours?	no<br>a little<br>rather much<br>very much	Hyo Jung Tak, Gavin W. Hougham, Atsuko Ruhnke, Gregory W. Ruhnke. The effect of in-office waiting time on physician visit frequency among working-age adults, 2014	2.8	2.7	2.5	2.6	2.7	2.8	2.7	2.6	2.6	2.7
Have you ensured that the Diagnosis/Therapy/Follow-up processes are adequately illustrated and explained to patients during the visit?	no<br>a little<br>rather much<br>very much	https://www.ahrq.gov/sites/default/files/wysiwyg/cahps/surveys-guidance/cg/cg-comparison-3-4.pdf	2.9	2.8	2.7	2.8	2.8	2.8	2.8	2.8	2.8	2.8
Did you arrange for the clinicians/nurses to answer the patient's and/or caregiver's questions comprehensively during the visit?	no<br>a little<br>rather much<br>very much	https://www.ahrq.gov/sites/default/files/wysiwyg/cahps/surveys-guidance/cg/cg-comparison-3-4.pdf	2.8	2.9	2.8	2.8	2.8	2.7	2.8	2.8	2.8	2.8
Did you ensure that during the visit the sharing of the care plan between all caregivers and with the patient is promoted?	no<br>a little<br>rather much<br>very much	Canberra Health Services. The Foundation for Exceptional Care Clinical Governance Framework 2020–2023	2.8	2.8	2.7	2.8	2.8	2.9	2.7	2.7	2.7	2.8
If yes, did you arrange for a record to be left on the Company Information System?	yes<br>no		2.6	2.5	2.7	2.6	2.6	2.7	2.4	2.6	2.6	2.6
Did you arrange for health professionals to provide information material on the disease and/or voluntary associations during the visit?	no<br>a little<br>rather much<br>very much		2.7	2.5	2.5	2.6	2.6	2.6	2.5	2.6	2.6	2.6
Does your company follow a standardised follow-up procedure (transmission of copies of documentation to the caregiver, booking a follow-up visit, etc.)?	no<br>a little<br>rather much<br>very much	Care Quality Commission. NHS Core Inpatient Survey 2021. Survey handbook. Ipsos MORI, Coordination Centre for Mixed Methods. Version 1.	2.6	2.7	2.6	2.6	2.6	2.7	2.7	2.6	2.7	2.7
Is there a Care Pathway (CP) related to the patient's health problem?	yes<br>no	Edward Abrahams, Alan Balch, Patricia Goldsmith, Marcia Kean, Amy M. Miller, Gilbert Omenn, Ellen Sonet, John Sprandio, Courtney Tyne, Kimberly Westrich; Clinical Pathways: Recommendations for Putting Patients at the Center of Value-Based Care. Clin Cancer Res 15 August 2017; 23 (16): 4545–4549. https://doi.org/10.1158/1078-0432.CCR-17-1609	3.0	2.9	2.8	2.9	2.9	2.9	3.0	3.0	2.9	3.0
Is it used to provide data on clinical performance related to the health problem?	no<br>a little<br>rather much<br>very much	Specchia, M.L., La Torre, G., Siliquini, R. et al. OPTIGOV - A new methodology for evaluating Clinical Governance implementation by health providers. BMC Health Serv Res 10, 174 (2010).	2.9	2.8	2.8	2.7	2.8	3.0	2.8	2.7	2.7	2.8
Has the company management put you in a position to organise opportunities to discuss performance and/or quality of care data (audits, M&M meetings, etc.)?	no<br>a little<br>rather much<br>very much	Specchia, M.L., La Torre, G., Siliquini, R. et al. OPTIGOV - A new methodology for evaluating Clinical Governance implementation by health providers. BMC Health Serv Res 10, 174 (2010).	2.8	2.8	2.7	2.7	2.8	2.9	2.8	2.8	2.7	2.8
Does the management foresee time for multidisciplinary discussion of the health problem (other than consultation)?	no<br>a little<br>rather much<br>very much	Specchia, M.L., La Torre, G., Siliquini, R. et al. OPTIGOV - A new methodology for evaluating Clinical Governance implementation by health providers. BMC Health Serv Res 10, 174 (2010).	2.9	2.8	2.7	2.7	2.8	3.0	2.8	2.7	2.8	2.8
Is provision made for updating/training/empowerment for you on continuous quality improvement issues?	yes<br>no	Specchia, M.L., La Torre, G., Siliquini, R. et al. OPTIGOV - A new methodology for evaluating Clinical Governance implementation by health providers. BMC Health Serv Res 10, 174 (2010).	2.7	2.6	2.6	2.6	2.6	2.8	2.7	2.7	2.7	2.7
Does your company use questionnaires to assess organisational well-being/work experience?	yes<br>no	Al-Abri R, Al-Balushi A. Patient satisfaction survey as a tool towards quality improvement. Oman Med J. 2014;29(1):3-7. doi:10.5001/omj.2014.02	2.8	2.6	2.7	2.7	2.7	2.7	2.6	2.7	2.7	2.7
SECTION: SAFETY			General relevance	Support from scientific evidence	Measurability	Actionability	OVERALL	General relevance	Support from scientific evidence	Measurability	Actionability	OVERALL
	SCORE:	SOURCE:										
Does the management provide for facilitated routes to the facility (outpatient clinic), also in relation to patients' care needs (parking, architectural barriers, signposting, information box, waiting room seating)?	no<br>a little<br>rather much<br>very much	Matin BK, Williamson HJ, Karyani AK, Rezaei S, Soofi M, Soltani S. Barriers in access to healthcare for women with disabilities: a systematic review in qualitative studies. BMC Womens Health. 2021;21(1):44. Published 2021 Jan 30. doi:10.1186/s12905-021-01189-5	2.8	2.5	2.6	2.6	2.6	2.7	2.6	2.6	2.6	2.6
Do you consider that all the conditions for the safe provision of services (physical, biological, environmental risks) e.g. needles, ICA, catheter-operator hand washing, fall risk, use of Personal Protective Equipment have been made available to you?	no<br>a little<br>rather much<br>very much	Canberra Health Services. The Foundation for Exceptional Care Clinical Governance Framework 2020–2023	2.9	2.7	2.7	2.7	2.8	3.0	2.8	2.7	2.8	2.8

Are risks and/or complications of procedures communicated by the treatment team according to standardised procedures?	no<br>a little<br>rather much<br>very much	Canberra Health Services. The Foundation for Exceptional Care Clinical Governance Framework 2020–2023	3.0	2.9	2.9	2.9	2.9	3.0	2.9	2.8	2.9	2.9
Have you given instructions for the communication of possible adverse reactions of prescribed drugs to the patient to be promoted during the visit?	no<br>a little<br>rather much<br>very much	Bomhof-Roordink H, Gärtner FR, van Duijn-Bakker N, van der Weijden T, Stiggelbout AM, Pieterse AH. Measuring shared decision making in oncology: Development and first testing of the iSHAREpatient and iSHAREphysician questionnaires. Health Expect. 2020 Apr;23(2):496-508. doi: 10.1111/hex.13015. Epub 2020 Feb 5. PMID: 32022350; PMCID: PMC7104639.	2.8	2.8	2.9	2.8	2.8	2.9	2.7	2.8	2.8	2.8
Have operational instructions been provided for risk management and control (aimed at reducing error) during the administration of therapy?	no<br>a little<br>rather much<br>very much	Robyn Clay-Williams et al. clinician safety culture and leadership questionnaire: refinement and validation in Australian public hospitals | International Journal for Quality in Health Care.	2.9	2.8	2.6	2.6	2.7	2.8	2.7	2.6	2.6	2.7
Does clinical risk management follow standardised procedures?	no<br>a little<br>rather much<br>very much	Laura L. Gleeson et al. Safety culture in a major accredited Irish university teaching hospital: a mixed methods study using the safety attitudes questionnaire.<br>Canberra Health Services. The Foundation for Exceptional Care Clinical Governance Framework 2020–2023	2.7	2.5	2.6	2.5	2.6	2.8	2.6	2.6	2.5	2.6
Does your company concretely pursue a culture of learning from errors (training, audits, reporting)?	no<br>a little<br>rather much<br>very much	Specchia, M.L., La Torre, G., Siliquini, R. et al. OPTIGOV - A new methodology for evaluating Clinical Governance implementation by health providers. BMC Health Serv Res 10, 174 (2010).	2.8	2.8	2.6	2.6	2.7	2.7	2.7	2.6	2.6	2.6
Does your management have policies/procedures in place to counter any climate of hostility and/or aggressiveness on the part of patients/health workers?	no<br>a little<br>rather much<br>very much		2.8	2.5	2.7	2.7	2.7	2.7	2.4	2.7	2.6	2.6

PERSPECTIVE: PATIENT			DELPHI: FIRST ROUND				OVERALL	DELPHI: SECOND ROUND				OVERALL
SECTION: QUALITY			General relevance	Support from scientific evidence	Measurability	Actionability		General relevance	Support from scientific evidence	Measurability	Actionability	
	SCORE:	SOURCE:										
Were you able to book the appointment for the visit easily (telephone, email, live)?	no<br>a little<br>rather much<br>very much	Zhao P, Yoo I, Lavoie J, Lavoie BJ, Simoes E. Web-Based Medical Appointment Systems: A Systematic Review. J Med Internet Res. 2017;19(4):e134. Published 2017 Apr 26. doi:10.2196/jmir.6747	2.7	2.6	2.6	2.7	2.6	2.8	2.5	2.6	2.7	2.7
Were the visiting times adhered to?	no<br>a little<br>rather much<br>very much	Hyo Jung Tak, Gavin W. Hougham, Atsuko Ruhnke, Gregory W. Ruhnke. The effect of in-office waiting time on physician visit frequency among working-age adults, 2014	2.8	2.5	2.7	2.6	2.7	2.8	2.5	2.9	2.8	2.8
Did you receive complete and comprehensible information on diagnosis/therapy/follow-up?	no<br>a little<br>rather much<br>very much	https://www.ahrq.gov/sites/default/files/wysiwyg/cahps/surveys-guidance/cg/cg-comparison-3-4.pdf	2.9	2.7	2.8	2.7	2.8	2.8	2.8	2.8	2.7	2.8
Was the visiting time devoted to you satisfactory for asking questions and receiving clarification?	no<br>a little<br>rather much<br>very much	https://www.ahrq.gov/sites/default/files/wysiwyg/cahps/surveys-guidance/cg/cg-comparison-3-4.pdf	2.8	2.5	2.7	2.6	2.7	2.9	2.6	2.7	2.6	2.7
Did the care team share the care plan with you?	no<br>a little<br>rather much<br>very much	Canberra Health Services. The Foundation for Exceptional Care Clinical Governance Framework 2020–2023	2.9	2.8	2.9	2.7	2.8	2.9	2.8	2.7	2.9	2.8
If yes, was the care plan formalised?	yes<br>no		2.9	2.6	2.8	2.8	2.8	2.8	2.5	2.9	2.8	2.8
During the visit, were you provided with information material on your pathology and/or voluntary associations?	no<br>a little<br>rather much<br>very much		2.7	2.5	2.6	2.5	2.6	2.6	2.4	2.5	2.5	2.5
Were there clear indications about follow-up appointments (email, telephone, contact person) in the documentation issued to you?	no<br>a little<br>rather much<br>very much	Care Quality Commission. NHS Core Inpatient Survey 2021. Survey handbook. Ipsos MORI, Coordination Centre for Mixed Methods. Version 1.	2.8	2.6	2.7	2.6	2.7	2.9	2.5	2.6	2.7	2.7
Were you informed of the existence of a clinical care pathway dedicated to your health problem?	yes<br>no	Edward Abrahams, Alan Balch, Patricia Goldsmith, Marcia Kean, Amy M. Miller, Gilbert Omenn, Ellen Sonet, John Sprandio, Courtney Tyne, Kimberly Westrich; Clinical Pathways: Recommendations for Putting Patients at the Center of Value-Based Care. Clin Cancer Res 15 August 2017; 23 (16): 4545–4549. https://doi.org/10.1158/1078-0432.CCR-17-1609	3.0	3.0	2.9	2.8	2.9	2.9	3.0	2.9	2.8	2.9
At the end of the visit, were you asked to complete a questionnaire on perceived quality?	yes<br>no	Al-Abri R, Al-Balushi A. Patient satisfaction survey as a tool towards quality improvement. Oman Med J. 2014;29(1):3-7. doi:10.5001/omj.2014.02	2.7	2.6	2.6	2.6	2.6	2.8	2.5	2.7	2.6	2.7
SECTION: SAFETY			General relevance	Support from scientific evidence	Measurability	Actionability	OVERALL	General relevance	Support from scientific evidence	Measurability	Actionability	OVERALL
	SCORE:	SOURCE:										
Were you comfortable accessing the facility, also in relation to your care needs (parking, architectural barriers, signposting, information box, waiting room seating)?	no<br>a little<br>rather much<br>very much	Matin BK, Williamson HJ, Karyani AK, Rezaei S, Soofi M, Soltani S. Barriers in access to healthcare for women with disabilities: a systematic review in qualitative studies. BMC Womens Health. 2021;21(1):44. Published 2021 Jan 30. doi:10.1186/s12905-021-01189-5	2.7	2.7	2.7	2.6	2.7	2.7	2.6	2.8	2.6	2.7
Do you consider that the service was performed safely (physical, biological, environmental risks) e.g. washing of hands by operators, risk of falling, use of Personal Protective Equipment?	no<br>a little<br>rather much<br>very much	Canberra Health Services. The Foundation for Exceptional Care Clinical Governance Framework 2020–2023	2.8	2.7	2.7	2.6	2.7	2.8	2.8	2.7	2.7	2.8
Did you understand the risks and/or complications of the medical/surgical treatment?	no<br>a little<br>rather much<br>very much	Canberra Health Services. The Foundation for Exceptional Care Clinical Governance Framework 2020–2023	2.8	2.9	2.8	2.7	2.8	2.9	2.9	2.7	2.7	2.8
If drugs were prescribed, were the possible adverse reactions explained to you?	no<br>a little<br>rather much<br>very much	Bomhof-Roordink H, Gärtner FR, van Duijn-Bakker N, van der Weijden T, Stiggelbout AM, Pieterse AH. Measuring shared decision making in oncology: Development and first testing of the iSHAREpatient and iSHAREphysician questionnaires. Health Expect. 2020 Apr;23(2):496-508. doi: 10.1111/hex.13015. Epub 2020 Feb 5. PMID: 32022350; PMCID: PMC7104639.	2.7	2.7	2.7	2.6	2.7	2.8	2.7	2.6	2.6	2.6

If you take several drugs, have you been explained the risks and/or complications of polypharmacotherapy?	no<br>a little<br>rather much<br>very much	Robyn Clay-Williams et al. clinician safety culture and leadership questionnaire: refinement and validation in Australian public hospitals | International Journal for Quality in Health Care.<br>Anthony R. Cox and Tehreem F. Butt. Adverse Drug Reactions When the Risk Becomes a Reality for Patients.	2.8	2.6	2.6	2.6	2.7	2.7	2.6	2.6	2.5	2.6
Did you perceive a climate of hostility and/or aggressiveness before or during the service?	no<br>a little<br>rather much<br>very much		2.8	2.5	2.6	2.6	2.6	2.7	2.5	2.6	2.6	2.6

PERSPECTIVE: CAREGIVERS			DELPHI: FIRST ROUND				OVERALL	DELPHI: SECOND ROUND				OVERALL
SECTION: QUALITY			General relevance	Support from scientific evidence	Measurability	Actionability		General relevance	Support from scientific evidence	Measurability	Actionability	
	SCORE:	SOURCE:										
Were you able to book the appointment for the visit easily (telephone, email, live)?	no<br>a little<br>rather much<br>very much	Zhao P, Yoo I, Lavoie J, Lavoie BJ, Simoes E. Web-Based Medical Appointment Systems: A Systematic Review. J Med Internet Res. 2017;19(4):e134. Published 2017 Apr 26. doi:10.2196/jmir.6747	2.7	2.7	2.6	2.7	2.7	2.8	2.6	2.7	2.7	2.7
Were the visiting times adhered to?	no<br>a little<br>rather much<br>very much	Hyo Jung Tak, Gavin W. Hougham, Atsuko Ruhnke, Gregory W. Ruhnke. The effect of in-office waiting time on physician visit frequency among working-age adults, 2014	2.8	2.6	2.8	2.6	2.7	2.7	2.7	2.7	2.8	2.7
Did you receive complete and comprehensible information on diagnosis/therapy/follow-up?	no<br>a little<br>rather much<br>very much	https://www.ahrq.gov/sites/default/files/wysiwyg/cahps/surveys-guidance/cg/cg-comparison-3-4.pdf	2.8	2.7	2.7	2.7	2.7	2.7	2.7	2.8	2.7	2.7
Was the visit time satisfactory for asking questions and receiving clarification?	no<br>a little<br>rather much<br>very much	https://www.ahrq.gov/sites/default/files/wysiwyg/cahps/surveys-guidance/cg/cg-comparison-3-4.pdf	2.7	2.6	2.6	2.6	2.6	2.8	2.5	2.6	2.6	2.6
During the visit, did the care team share the care plan with you?	no<br>a little<br>rather much<br>very much	Canberra Health Services. The Foundation for Exceptional Care Clinical Governance Framework 2020–2023	2.8	2.8	2.8	2.7	2.8	2.8	2.8	2.7	2.7	2.8
If yes, was the care plan formalised?	yes<br>no		2.8	2.7	2.8	2.7	2.8	2.8	2.6	2.7	2.8	2.7
During the visit, were you provided with information material on your pathology and/or voluntary associations?	no<br>a little<br>rather much<br>very much		2.7	2.5	2.6	2.5	2.6	2.6	2.4	2.5	2.5	2.5
Were there clear indications about follow-up appointments (email, telephone, contact person) in the documentation issued to you?	no<br>a little<br>rather much<br>very much	Care Quality Commission. NHS Core Inpatient Survey 2021. Survey handbook. Ipsos MORI, Coordination Centre for Mixed Methods. Version 1.	2.8	2.6	2.7	2.6	2.7	2.9	2.5	2.6	2.7	2.7
Were you informed of the existence of a clinical care pathway dedicated to your health problem?	yes<br>no	Edward Abrahams, Alan Balch, Patricia Goldsmith, Marcia Kean, Amy M. Miller, Gilbert Omenn, Ellen Sonet, John Sprandio, Courtney Tyne, Kimberly Westrich; Clinical Pathways: Recommendations for Putting Patients at the Center of Value-Based Care. Clin Cancer Res 15 August 2017; 23 (16): 4545–4549. https://doi.org/10.1158/1078-0432.CCR-17-1609	3.0	3.0	2.9	2.8	2.9	2.9	3.0	2.9	2.8	2.9
At the end of the visit, were you asked to complete a questionnaire on perceived quality?	yes<br>no	Al-Abri R, Al-Balushi A. Patient satisfaction survey as a tool towards quality improvement. Oman Med J. 2014;29(1):3-7. doi:10.5001/omj.2014.02	2.7	2.5	2.7	2.6	2.6	2.8	2.6	2.6	2.6	2.7
SECTION: SAFETY			General relevance	Support from scientific evidence	Measurability	Actionability	OVERALL	General relevance	Support from scientific evidence	Measurability	Actionability	OVERALL
	SCORE:	SOURCE:										
Were you comfortable accessing the facility, also in relation to your care needs (parking, architectural barriers, signposting, information box, waiting room seating)?	no<br>a little<br>rather much<br>very much	Matin BK, Williamson HJ, Karyani AK, Rezaei S, Soofi M, Soltani S. Barriers in access to healthcare for women with disabilities: a systematic review in qualitative studies. BMC	2.8	2.5	2.7	2.6	2.7	2.7	2.6	2.6	2.6	2.6
Do you consider that the service was performed safely (physical, biological, environmental risks) e.g. washing of hands by operators, risk of falling, use of Personal Protective Equipment?	no<br>a little<br>rather much<br>very much	Canberra Health Services. The Foundation for Exceptional Care Clinical Governance Framework 2020–2023	2.9	2.7	2.6	2.6	2.7	2.8	2.9	2.7	2.6	2.7
Did you understand the risks and/or complications of the medical/surgical treatment?	no<br>a little<br>rather much<br>very much	Canberra Health Services. The Foundation for Exceptional Care Clinical Governance Framework 2020–2023	2.9	2.8	2.8	2.7	2.8	2.9	2.9	2.7	2.7	2.8
In the case of prescription drugs, have the possible adverse reactions been explained?	no<br>a little<br>rather much<br>very much	Bomhof-Roordink H, Gärtner FR, van Duijn-Bakker N, van der Weijden T, Stiggelbout AM, Pieterse AH. Measuring shared decision making in oncology: Development and first testing of	2.7	2.8	2.6	2.6	2.7	2.7	2.7	2.7	2.6	2.7

In the case of taking several drugs, have the risks and/or complications of polypharmacotherapy been explained?	no<br>a little<br>rather much<br>very much	Robyn Clay-Williams et al. clinician safety culture and leadership questionnaire: refinement and validation in Australian public hospitals | International Journal for Quality in	2.9	2.7	2.6	2.5	2.7	2.8	2.7	2.5	2.4	2.6
Did you perceive a climate of hostility and/or aggressiveness before or during the service?	no<br>a little<br>rather much<br>very much		2.8	2.6	2.7	2.6	2.7	2.8	2.5	2.6	2.7	2.7
